# The dual inhibitory effect of thiostrepton on FoxM1 and EWS/FLI1 provides a novel therapeutic option for Ewing’s sarcoma

**DOI:** 10.3892/ijo.2013.2016

**Published:** 2013-07-12

**Authors:** ANIRUDDHA SENGUPTA, MAHBUBUR RAHMAN, SILVIA MATEO-LOZANO, OSCAR M. TIRADO, VICENTE NOTARIO

**Affiliations:** 1 Department of Radiation Medicine, Molecular Oncology Program, Lombardi Comprehensive Cancer Center, Georgetown University Medical Center, Washington, DC 20057, USA; 2 Developmental Tumor Biology Laboratory, Hospital Sant Joan de Déu and Fundació Sant Joan de Déu, Barcelona, Spain; 3 Institut d’Investigació Biomèdica de Bellvitge (IDIBELL), Laboratori d’Oncología Molecular, L’Hospitalet de Llobregat, Barcelona, Spain

**Keywords:** Ewing’s sarcoma, FoxM1, cell cycle, EWS/FLI1, apoptosis, therapy

## Abstract

The poor prognosis of Ewing’s sarcoma (EWS), together with its high lethal recurrence rate and the side-effects of current treatments, call for novel targeted therapies with greater curative effectiveness and substantially reduced side-effects. The oncogenic chimeric protein EWS/FLI1 is the key malignancy driver in most EWSs, regulating numerous target genes, many of which influence cell cycle progression. It has often been argued that targeting proteins regulated directly or indirectly by EWS/FLI1 may provide improved therapeutic options for EWS. In this context, our study examined FoxM1, a key cell cycle regulating transcription factor, reported to be expressed in EWS and influenced by EWS/FLI1. Thiostrepton, a naturally occurring small molecule, has been shown to selectively inhibit FoxM1 expression in cancer cells. We demonstrate that in EWS, in addition to inhibiting FoxM1 expression, thiostrepton downregulates the expression of EWS/FLI1, both at the mRNA and protein levels, leading to cell cycle arrest and, ultimately, to apoptotic cell death. We also show that thiostrepton treatment reduces the tumorigenicity of EWS cells, significantly delaying the growth of nude mouse xenograft tumors. Results from this study demonstrate a novel action of thiostrepton as inhibitor of the expression of the EWS/FLI1 oncoprotein *in vitro* and *in vivo*, and that it shows greater efficacy against EWS than against other tumor types, as it is active on EWS cells and tumors at concentrations lower than those reported to have effective inhibitory activity on tumor cells derived from other cancers. Owing to the dual action of this small molecule, our findings suggest that thiostrepton may be particularly effective as a novel agent for the treatment of EWS patients.

## Introduction

Ewing’s sarcoma, a cancer predominantly affecting children and young adults, is a highly aggressive disease, with 25–30% patients exhibiting metastases at diagnosis (1–3). The rate of lethal recurrence is high in EWS patients and despite multimodal therapeutic regimens combining intensive treatments with chemotherapy, enhanced surgical procedures, and advanced radiotherapy, the 5-year disease-free survival rate for patients with localized EWS is only 60–70%, while for those with metastatic disease it drops down to 30% (2,4). The poor prognosis of EWS in addition to long-term side-effects of chemotherapy and radiation warrant the need for novel targeted therapies, which would minimize or eliminate side-effects including developmental anomalies, elevated risk of recurrence, or increased incidence of secondary malignancies.

EWS is cytogenetically characterized by the presence of a reciprocal translocation t(11;22)(q24;q12), that generates chimeric genes resulting from joining the 5′ half of the *EWS* gene and the 3′ half of a gene belonging to the *ETS* family of transcription factors, most frequently the *FLI1* gene. These hybrid *EWS/FLI1* genes, which are present in about 90% of EWS cases, are heterogeneous with regard to the location of the translocation junction, having different breakpoints (1). Nevertheless, regardless of the fusion type, all EWS/FLI1 proteins act as aberrant transcription factors that are responsible for the highly malignant phenotype and poor prognosis for EWS (1). The transcriptional activation domain of EWS and the DNA binding domain of FLI1 enable the EWS/FLI1 protein to regulate numerous target genes involved in various cellular functions, some of which are critical for the development of EWS (5,6). Gene expression profiling demonstrated that many regulated genes are indirect targets of EWS/FLI1, suggesting that EWS/FLI1-mediated oncogenesis results from direct and indirect mechanisms (7–9). A key characteristic of transformed cells is their alteration of cell proliferation (10), with deregulation of genes involved in controlling cell cycle being often the cause for unrestrained proliferation, ultimately leading to oncogenesis (11). The oncogenic nature of EWS/FLI1 makes it a putative candidate to alter the cell cycle, either directly or indirectly, to promote EWS growth (12). A recent study using both patient-derived cell lines and tumor samples from individual EWS patients demonstrated that many EWS/FLI1 upregulated genes do have roles in cell cycle control (13).

The forkhead box (Fox) proteins are evolutionarily conserved transcription factors possessing a conserved DNA-binding domain (DBD), or forkhead box. These proteins, which have been grouped into 19 families (Fox A-S), are involved in various cellular processes including growth and differentiation, embryogenesis, metabolism, development, apoptosis, migration and invasion (14). Due to the importance of Fox proteins not only in development, but also in adult organisms, a loss or gain of function for *Fox* genes can impact cell fate and development. It is therefore not surprising that deregulation of *Fox* genes have been reported in several genetic disorders as well as in cancer (14,15). Gene expression studies involving *EWS/FLI1* knockdown in EWS samples have shown regulation of *Fox* genes, with *FoxM1* being significantly downregulated upon knockdown of EWS/FLI1 (13,16). FoxM1 has a well characterized role in cell cycle progression through regulation of the G1/S and G2/M phases of cell cycle (17,18), but has also been characterized as an oncogene, with aberrant expression in a variety of cancers (19–25). FoxM1 has been proposed to be involved with uncontrolled cell division in early stages of tumorigenesis (21,26,27), and increased expression of FoxM1 remarkably correlates with progressive cancer stages (20,23,24,27). Thiostrepton, a natural product with antibiotic properties isolated from *Streptomyces azureus*, is known to inhibit the binding of FoxM1 to genomic target sites (28) and cellular studies showed that thiostrepton selectively targets breast cancer cells by inhibiting FoxM1 expression (29).

It has been recently reported that FoxM1 expression is elevated in EWS and that thiostrepton treatment reduced the viability of cultured EWS cell lines in a dose-dependent manner (30). However, its effect on tumor xenografts remained to be determined. Herein, we demonstrate that thiostrepton not only influenced FoxM1 expression in EWS, but also inhibited the expression of the EWS/FLI1 oncoprotein, reducing both their mRNA and protein levels. Thiostrepton exposure of EWS cells in culture caused their apoptotic cell death at doses much lower than those reported to cause similar effects against other cancer cell types. Moreover, thiostrepton treatment reduced the tumorigenicity of EWS cells, significantly delaying tumor growth in nude mice xenografts. Collectively, these data strongly suggest that thiostrepton may be used to simultaneously target EWS/FLI1 and FoxM1 in EWS cells and potentially be developed as a novel anticancer drug with enhanced efficacy against EWS.

## Materials and methods

### Reagents

DMEM, trypsin and antibiotics were purchased from Mediatech Inc. (Manassas, VA). The ECL western blotting substrate was purchased from Thermo Fisher Scientific Inc. (Waltham, MA); Alexa Fluor 488-conjugated secondary antibodies and oligonucleotides were from Invitrogen (Carlsbad, CA); the protease inhibitor cocktail was from Roche Applied Science (Indianapolis, IN); CellTiter-Glo Luminescent Cell Viability Assay and Caspase 3/7 assay kits were procured from Promega (Madison, WI); Matrigel™ basement membrane matrix was from BD Biosciences (Franklin Lakes, NJ); ethidium homodimer (EthD-1) was from Cell Biolabs Inc. (San Diego, CA), and thiostrepton was procured from EMD-Millipore (Billerica, MA). Antibodies against FoxM1, Fli1 and actin were from Santa Cruz Biotechnology Inc. (Dallas, TX); antibodies against cyclin D1, PLK1, Akt, PARP, cleaved caspase 7, and survivin were from Cell Signaling Technology (Danvers, MA); the antibody against XIAP was from BD Bioscience; the anti-Src antibody was from Epitomics Inc. (Burlingame, CA); and anti-Cep55 was from Abnova (Jhongli City, Taiwan, R.O.C.). Unless otherwise mentioned, chemicals used were of molecular biology or cell culture grade and were procured from Thermo Fisher Scientific Inc.

### Cell lines, culture, treatment and viability assays

The EWS cell lines (A4573, SK-ES-1 and TC-71) and the breast cancer cell line (MCF-7) were maintained in DMEM supplemented with antibiotics and 10% fetal bovine serum. Cells were incubated at 37°C in a humidified chamber with 5% CO_2_. Cells treated with thiostrepton (dissolved in DMSO) for 48 h were harvested and processed for experiments. Cell viability was measured using the CellTiter-Glo Luminescent Cell Viability Assay kit (Promega) in a 24- or 96-well plate format, according to the manufacturer’s protocol. Following growth for the selected time points, luminescence intensity measured for each sample was plotted relative to values obtained for control cells. Data represent the average from at least three independent experiments.

### Cell cycle analysis

EWS cells were harvested 48 h after exposure to thiostrepton, washed once in PBS, fixed in 70% ethanol in PBS, and stained with propidium iodide. Flow cytometric analysis was carried out on a FACScan instrument (Becton-Dickinson, San Jose, CA), performed at the Flow Cytometry/Cell Sorting Shared Resource of the Lombardi Comprehensive Cancer Center.

### Reverse transcription-PCR

RNA extraction, reverse transcription-PCR (RT-PCR) conditions, and PCR product analysis were carried out as described (31). Primer pairs 5′-ACAAGC CCAACAACAAGG-3′ (forward) and 5′-ATCGGGATGCCA AAGAGG-3′ (reverse) were used to amplify EWS/FLI-1, while 5′-GCCAACCGCTACTTGACATT-3′ (forward) and 5′-TCT CCTCTTTCCCTGGTCCT-3′ (reverse) were used for FoxM1. Tubulin was used as internal control, with the primer set 5′-GCAGATGCTTAACGTGCAGA-3′ (forward) and 5′-GGCA TCCTGGTACTGCTGAT-3′ (reverse). The number of cycles for PCR was adjusted to ensure that the reaction end-points remained within the exponential phase of product amplification, to get a semi-quantitative estimate of relative mRNA abundance.

### Tumorigenicity assays

Tumorigenic activity was determined through xenograft assays carried out as previously described (31). Male immunodeficient, athymic nude (BALB/c nu/nu) mice were obtained from Harlan Laboratories (Indianapolis, IN) and all animal work was done under protocols approved by the Georgetown University Animal Care and Use Committee. EWS cells (5×10^6^ cells in 100 μl of serum-free medium) were mixed with 100 μl of Matrigel basement membrane matrix and injected s.c. into the posterior flank of mice. Once tumors reached a mean volume of about 150 mm^3^, mice were divided into control and treatment groups (10 animals per group). Treatment groups received thiostrepton (given i.p. at a dose of 17 mg/kg), while control groups received i.p. injections of thiostrepton carrier solution (32) in equal volume. Animals were injected every third day from the initial injection of thiostrepton and tumors were measured every alternate day. At specified times during the treatment, or whenever tumors reached the maximum volume allowed by institutional tumor burden guidelines, the corresponding animals were sacrificed by asphyxiation with CO_2_ and tumors were excised for experimental purposes. Primary tumor volumes were calculated by the formula V = (1/2)a × b^2^, where a is the longest tumor axis and b is the shortest tumor axis. Data were plotted as mean values ± SE in quantitative experiments.

### Western blot analysis

Cells were lysed with RIPA buffer containing protease inhibitors, and lysates were centrifuged at 10,000 × g at 4°C for 15 min. Tissues were first homogenized in RIPA buffer containing protease inhibitors, and lysates were centrifuged at 10,000 × g at 4°C for 15 min. Lysates were subject to SDS-PAGE and the resolved polypeptides were transferred to PVDF membranes. Following transfer, the membranes were blocked with 5% skim-milk in PBST (PBS containing 0.1% Tween-20) at room temperature for 1 h, and incubated overnight with gentle rocking at 4°C with designated primary antibodies. Next, blots were washed with PBST and incubated at room temperature for 1 h with horseradish peroxidase (HRP)-conjugated secondary antibody, and the peroxidase activity was detected by enhanced chemiluminescence, using the ECL western blotting substrate.

### Immunofluorescence analyses

For fluorescence studies, the control and treated cells were cultured on cover-slips, washed once in PBS and fixed with 4% paraformaldehyde in PBS for 20 min at room temperature. Following fixation, the cells were washed in PBS and permeabilized with 0.2% Triton X-100 for 10 min. Blocking was done in 10% normal goat serum (Vector Laboratories, Burlingame, CA) in PBS for 30 min and incubated with FoxM1 (1:500 dilution) antibody for 1 h at room temperature. Cells were then washed twice in PBS for 10 min each, and incubated with Alexa fluor 488 conjugated anti-rabbit IgG for 1 h at room temperature. The cells were washed twice in PBS for 10 min each, followed by DNA staining with DAPI for 5 min, and finally washed once in PBS and once in deionized water before being mounted in Fluoro-Gel mounting medium (Electron Microscopy Sciences, Hatfield, PA) and viewed under a fluorescence microscope. Images were processed with ImageJ software.

### Caspase activity assay

Caspase 3/7 activity in the treated cells was determined using the Caspase 3/7 assay kit from Promega, according to the manufacturer’s protocol.

### Statistical analysis

Unless otherwise indicated, RT-PCR and western blot analyses were repeated at least thrice. Data from densitometric quantification analyses were expressed as mean ± SD. For these and other assays involving statistical analysis, ANOVA or Student’s t-tests were used to assess the significance of differences between groups or individual variables, respectively. p≤0.01 was regarded as significant.

## Results

### Thiostrepton inhibits the proliferation of EWS cells

**Figure 1:**
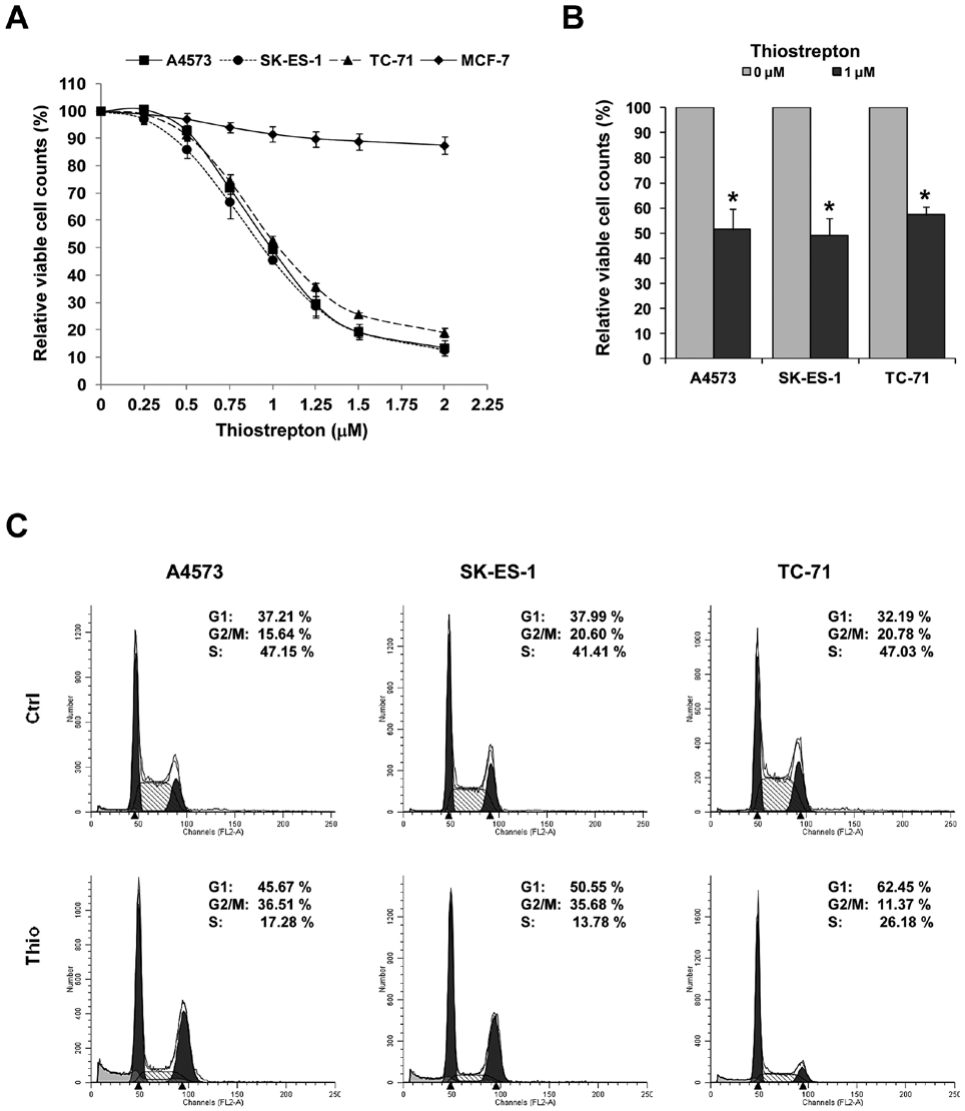
Thiostrepton influences cell proliferation and induces cell cycle arrest in EWS cells. (A) The effect of increasing concentrations of thiostrepton on the proliferation of EWS cells (A4573, SK-ES-1 and TC-71) and breast cancer MCF-7 cells was determined by CellTiter-Glo Luminescent Cell Viability assay kit in a 24-well plate format, according to the manufacturer’s protocol (n=3). (B) Viable cell counts of EWS cells after 48-h treatment with 1 μM thiostrepton; histogram bars represent mean percentages of the viable cell counts; bars, SD (^*^p≤0.0001; n=4). (C) EWS cells were treated with 1 μM thiostrepton for 48 h and cell cycle analysis was done following propidium iodide staining (n=3). Ctrl and Thio correspond to control and thiostrepton-treated cells, respectively.

**Figure 2:**
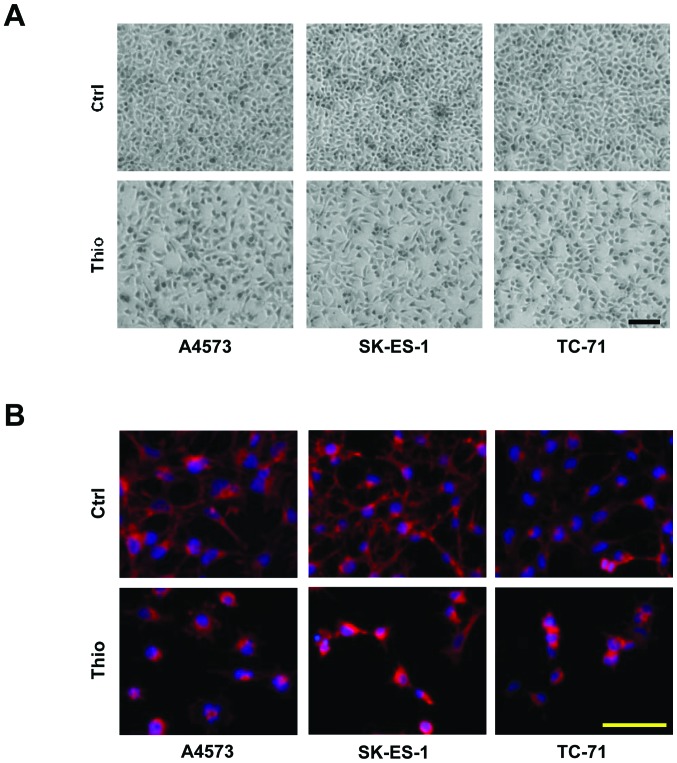
Altered morphology with disrupted actin network in thiostrepton treated EWS cells. (A) Phase contrast micrographs from control and cells treated with 1 μM thiostrepton for 48 h, showing altered morphology for treated cells (bottom panels); scale bar, 100 μm. (B) Treatment with thiostrepton alters the actin cytoskeleton in EWS cells; images represent rhodamine-labeled phalloidin staining for actin and nuclear staining with DAPI for the indicated cell lines. Scale bar, 100 μm. Ctrl and Thio correspond to control and thiostrepton-treated cells, respectively.

Thiostrepton, a natural compound, has been shown to exert anti-proliferative effects against human cancer cells, mainly through inhibition of FoxM1 activity (28). In order to determine the efficacy of this compound on EWS cells, we examined dose-dependent effects of thiostrepton on the proliferation and/or viability of EWS cell lines A4573, SK-ES-1 and TC-71, selected as representative of the three major EWS/FLI1 translocation types (1). MCF-7, a breast carcinoma cell line in which the effects of thiostrepton had been already characterized (29), was used as a control. Cells were examined up to 48 h after treatment with various doses of thiostrepton. While, as expected, MCF-7 cells did not show substantial changes in morphology or viable cell counts following treatment with up to 2 μM thiostrepton, the three EWS cell lines were clearly susceptible to thiostrepton treatment (Fig. 1A), with viable cell numbers decreasing in all cases by ~50% upon treatment with 1 μM thiostrepton (Fig. 1B). Consequently, unless otherwise indicated, EWS cells were treated with 1 μM thiostrepton for 48 h for all subsequent experiments. FACS analyses showed that cell cycle progression was altered in the cultures of the three treated cell lines, with a decrease in the percentage of the cell populations traversing through S-phase and the accumulation of cells in either the G1 or the G2-M phase (Fig. 1C). Thiostrepton also caused marked morphological changes. A large proportion of treated cells showed a round morphology and detached from plates, while those still adhering to plates showed a spread out polygonal shape, unlike their control counterparts, which were rather rounded and smaller (Fig. 2A). Thiostrepton also induced a change in the organization of actin cytoskeleton in EWS cells, with an intense staining observed near the nuclear periphery of treated cells (Fig. 2B).

### Thiostrepton inhibits the mRNA and protein expression of both FoxM1 and EWS/FLI1

**Figure 3:**
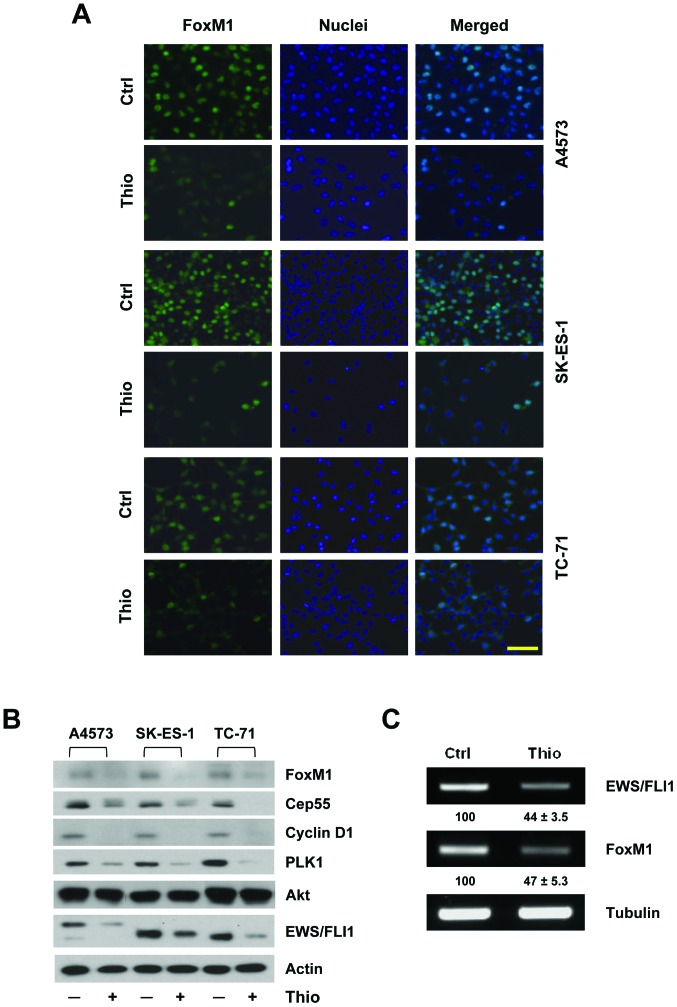
Thiostrepton reduces mRNA and protein expression of FoxM1 and EWS/FLI1. (A) Immunodetection of FoxM1 in thiostrepton-treated and control EWS cells. The image shows FoxM1 immunostaining (left column), nuclear staining with DAPI (center column) and their merged image (right column), for the cell lines indicated; scale bar, 100 μm; Ctrl and Thio correspond to control and thiostrepton-treated cells, respectively. (B) Immunoblot showing substantial reduction in levels of FoxM1 and some of its known downstream targets as well as in the levels of the EWS/FLI1 oncoprotein in thiostrepton-treated cells (+) relative to untreated (−) controls; β-actin was used as the loading control. (C) Thiostrepton treatment decreased the mRNA expression of FoxM1 and EWS/FLI1 in EWS cells relative to untreated controls, as detected through RT-PCR analysis; relative values for three independent experiments showing mean ± SD are depicted. Ctrl and Thio correspond to control and thiostrepton-treated cells, respectively.

Immunofluorescent detection revealed that FoxM1, which is predominantly localized in the nuclei of EWS cells, was substantially decreased when cells were treated with thiostrepton (Fig. 3A). This result was corroborated by western immunoblot analysis, which showed a significant decrease in FoxM1 expression (Fig. 3B). Accordingly, the expression levels of known FoxM1 downstream targets such as polo like kinase-1 (PLK-1), the 55-kDa centromeric protein Cep55, and cyclin D1 were also decreased following thiostrepton treatment. It is noteworthy that thiostrepton treatment did not induce any significant change in the expression of Akt, a known regulator of FoxM1 expression (29). We also investigated the possible effect of thiostrepton treatment on the expression of EWS/FLI1, the key oncogenic protein driving EWS malignant progression. Interestingly, the levels of the EWS/FLI1 oncoprotein were also markedly reduced in the treated cells (Fig. 3B), prompting us to examine if the levels of *EWS/FLI1* mRNA were altered upon thiostrepton treatment. RT-PCR analyses following thiostrepton treatment for 48 h revealed that transcript levels of both *FoxM1* and *EWS/FLI1* were substantially diminished relative to those in untreated controls (Fig. 3C), indicating that thiostrepton treatment decreases the expression of both mRNA and protein not only of FoxM1, but also, and most importantly, of EWS/FLI1 in EWS cells.

### Thiostrepton treatment inhibits the growth of EWS-derived tumors in vivo

**Figure 4:**
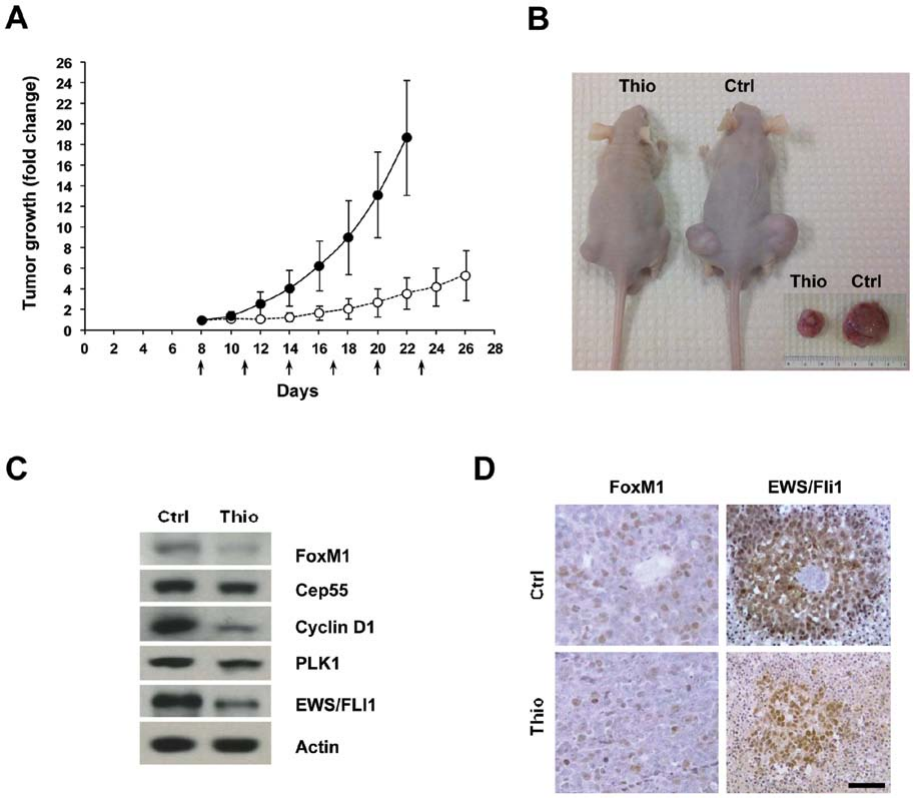
Thiostrepton treatment reduces the tumorigenicity of EWS cells. (A) Xenografts established by s.c. injection of A4573 cells in athymic (BALB/c nu/nu) nude mice were given i.p. dosage of thiostrepton (17 mg/kg) (n=10). Tumor growth in the animals was monitored on alternate days following s.c. cell injections, and statistically significant differences in tumor volume were noted for the treated sample around 22 days post-injection (p≤0.00002). Arrows indicate days when the mice were subjected to thiostrepton injection. (B) Mice injected with A4573 cells and treated with vehicle or with thiostrepton were euthanized when tumors in control animals reached maximum allowable tumor burden (22 days), and tumors were excised and their volume measured. Inset shows images of representative tumors isolated at 22 days post s.c. cell injection. (C) Immunoblot showing the reduction in the levels of FoxM1 and some of its transcriptional targets as well as of the EWS/FLI1 oncoprotein in lysates from mice treated with thiostrepton relative to those from untreated animals; β-actin was used as loading control. (D) Sections of xenograft tumors excised from mice treated with vehicle or triostrepton were subjected to immunohistochemical analysis to detect FoxM1 or EWS/FLI1. Results showed decreased staining for both target proteins in tumors excised from thiostrepton-treated mice relative to those obtained from control animals. Scale bar, 50 μm. Ctrl and Thio represent control and thiostrepton-treated samples.

The effects of thiostrepton on tumorigenicity were examined *in vivo* by s.c. injection of A4573 cells into nude mice and monitoring tumor growth. When tumors reached a volume of ~150 mm^3^ (around day 8) animals were randomized into two groups (n=10) and treated as described in Materials and methods. Subsequently, tumor growth was followed over a period of four weeks, measuring the volume every other day. Halfway through the treatment plan (day 16 after cell injections), tumor volumes in control mice had increased ~6-fold from the initiation of treatment, while their thiostrepton-treated counterparts increased only ~1.7-fold, exhibiting a ~3.5-fold reduction (p≤0.0005), relative to controls (Fig. 4A). Around 22 days after the s.c. cell injections, when animals from control groups had to be sacrificed following Institutional Animal Care and Use guidelines on maximum allowable tumor burden, the thiostrepton-treated tumors showed a significant decrease in size (Fig. 4B). Following sacrifice, the tumors were excised and their volumes determined. Measurements showed that tumors derived from control untreated cells increased ~19-fold in comparison to their volume at the start of the treatment, while those derived from cells treated with thiostrepton increased only ~4-fold, thus showing a ~4.75-fold difference in volume relative to the untreated controls (Fig. 4B inset). These data revealed that thiostrepton significantly hampers the growth of EWS tumors.

To examine whether the antitumor effects of thiostrepton treatment correlated with the expression levels of FoxM1, its downstream targets, and/or EWS/FLI1, we investigated these proteins by western immunoblot analysis of lysates prepared from xenograft tumors excised from control and treated animals. The data obtained agreed with our previous observations upon thiostrepton treatment of EWS cells, showing a decrease in the levels of FoxM1 and its downstream targets, as well as diminished levels of EWS/FLI1 in lysates from thiostrepton-treated tumors (Fig. 4C). Immunostaining for FoxM1 and EWS/FLI1 in paraffin-embedded sections of xenograft tumors show a decreased expression for both proteins in sections of tumors from thiostrepton-treated mice (Fig. 4D). Taken together, results indicated that the observed effects were a consequence of thiostrepton treatment, leading to decreased tumorigenicity of EWS cells.

### Thiostrepton inhibitory effects on EWS proliferation and tumorigenicity are mediated by a caspase 7-dependent apoptotic response

**Figure 5:**
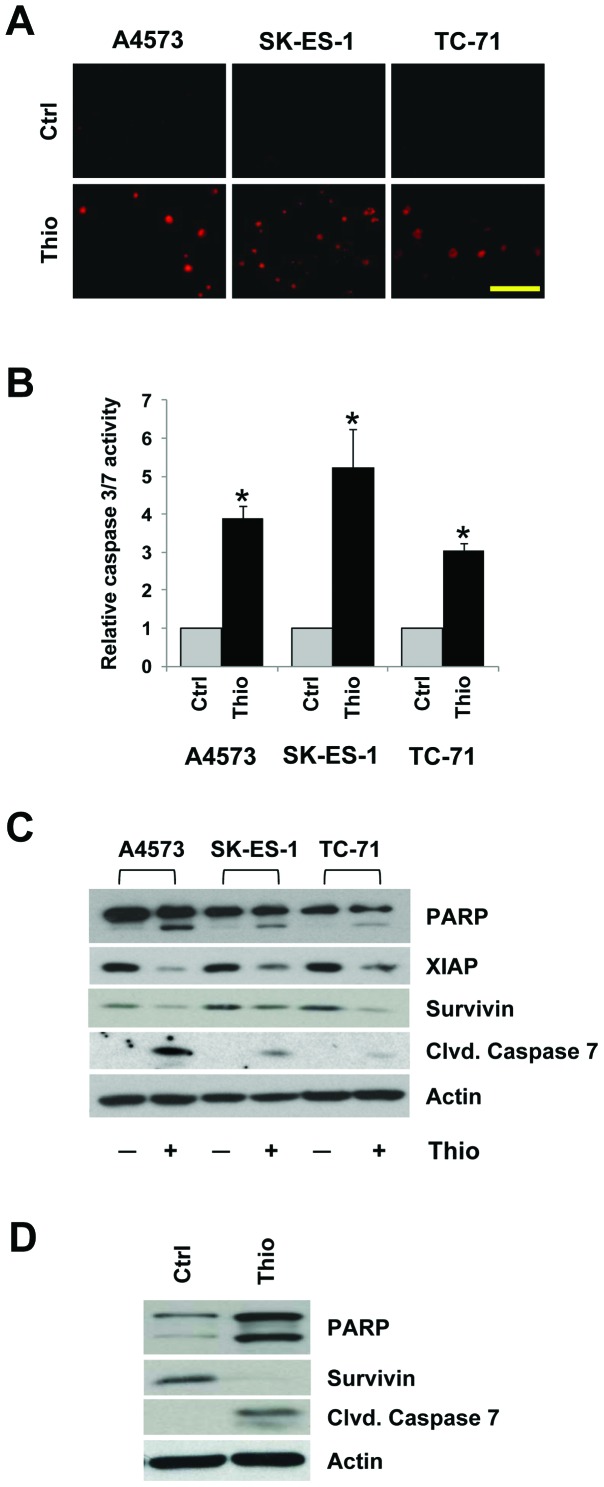
Thiostrepton induces a caspase 7-dependent apoptotic response in EWS cells and tumors. (A) Detection of cell death in thiostrepton-treated EWS cells, using the cell viability indicator ethidium homodimer-1 (EthD-1); scale bar, 100 μm. (B) Determination of caspase 3/7 activity in control and thiostrepton-treated EWS cells; histogram bars represent mean fold changes in activity; bars, SD (^*^p≤0.003; n=3). (C) Immunoblot showing cleavage of PARP, reduction in XIAP and survivin, along with the detection of cleaved-caspase 7 in lysates from control cells (−) and from thiostrepton-treated cells (+); β-actin was used as the loading control. (D) Immunoblot showing cleaved PARP, decreased survivin, and increased cleaved-caspase 7 in lysates from tumors derived from vehicle- or thiostrepton-treated animals; β-actin was used as the loading control. Wherever applicable, Ctrl and Thio represent control and thiostrepton-treated cells or animals, respectively.

In addition to the effects on cell cycle described above, thiostrepton treatment appeared to induce a dose-dependent extent of cell death, in agreement with the increased proportion of cells detected in the sub-G1 peak in thiostrepton-treated cultures. Consequently, an initial assessment of cell death was made by using EthD-1 staining of cells in culture. These examinations revealed that the observed changes in morphology were accompanied by the induction of apoptosis (Fig. 5A). As thiostrepton has been described as an activator of caspases (29), we determined the levels of caspase 3/7 activity in thiostrepton-treated cells and in the untreated controls. Results showed a 3- to 6-fold elevation in caspase activity in all thiostrepton-treated EWS cells (Fig. 5B). Consistent with the observed increase in caspase activity, western blot analyses revealed that thiostrepton caused PARP cleavage, decreased expression of XIAP and survivin, and the cleavage of caspase 7 in treated EWS cells (Fig. 5C) as well as in lysates of treated xenograft tumors (Fig. 5D), thus substantiating the results *in vitro* and *in vivo*. Although the *in vitro* caspase activity assays used did not distinguish between changes due to caspase 3 or caspase 7, the fact that cleaved caspase 3 could not be detected in samples of cells or tumors treated with thiostrepton using either western immunoblots, immunofluorescence or immunohistochemistry, supports the notion that thiostrepton induces the apoptotic death of EWS cells in culture and in xenograft tumors predominantly through a caspase 7-dependent mechanism.

## Discussion

The genes regulated by the aberrant transcription factor EWS/FLI1 play crucial roles in oncogenesis and EWS development (12,33). EWS/FLI1-regulated proteins have been frequently regarded as a source of potential therapeutic options for EWS patients. Gene expression studies involving EWS/FLI1 knockdown in EWS cells showed regulation of *Fox* genes, with *FoxM1* expression being significantly decreased after EWS/FLI1 downregulation (13,16). FoxM1 is involved in cell proliferation, being expressed only in actively cycling cells under normal conditions, and regulating the expression a number of genes involved in cell cycle control (18,34,35). In addition to its involvement in uncontrolled cell division during early stages of tumorigenesis (21,26,27), elevated levels of FoxM1 have been found associated with progressive stages of increasing malignancy in a variety of cancers (20,23,24,27). As overexpression of FoxM1 has been reported in more than 20 types of human cancer and is involved in multiple landmarks of cancer (36), the inhibition of FoxM1 activity is emerging as an attractive target for cancer therapy. Results in the current study provide strong evidence supporting the notion that the inhibition of FoxM1 is a possible therapeutic option for EWS patients.

Thiostrepton, a thiazole antibiotic, has been already identified as having anticancer activity (37), and a number of studies have demonstrated its cytotoxic effect through selective inhibition of FoxM1 on a wide variety of cancer cell lines (29,38,39). We studied the effect of thiostrepton on EWS cells and observed a better efficacy under identical conditions on EWS lines in comparison to a breast cancer cell line, for which the effect of thiostrepton had been previously characterized (Fig. 1A). Inhibition of FoxM1 altered cell cycle in EWS cell lines (Fig. 1C), corroborating the role of FoxM1 in cell cycle progression through G1/S and G2/M phases. The morphology of EWS cells was altered upon treatment with thiostrepton, manifested by their spontaneous detachment from the culture plates and by the modification in their actin cytoskeleton (Fig. 2). The altered actin organization may facilitate cell detachment, ultimately leading to cell death (40).

Loss of FoxM1 following thiostrepton treatment led to a concomitant decrease in the expression levels of proteins regulated by FoxM1, including PLK1, Cep55 and cyclin D1. However, proteins influencing FoxM1 expression were not changed, proving that the observed thiostrepton effects were due to alteration of signaling pathway(s) downstream of FoxM1 (Fig. 3), with one remarkable exception. Interestingly, the mRNA and protein levels of the EWS/FLI1 oncoprotein were reduced upon treatment with thiostrepton (Fig. 3). The reduction in mRNA expression suggests that this regulation might be at the transcriptional level. Since it was previously reported that thiostrepton inhibits FoxM1 binding to its genomic target sites (28), we hypothesized that the small molecule might be associated in a similar interfering mechanism with EWS/FLI1, leading to a reduction of the aberrant transcription factor. However, as our analysis of the 5′ regulatory sequences (up to 3,000 bp upstream of transcription initiation site) of the *EWS* gene indicated the absence of the major FoxM1 DNA-binding sites described to date (41,42), it seems that the effect of FoxM1 on the levels of *EWS/FLI1* mRNA may be the result of an indirect mechanism. Although it is possible that such a mechanism may involve the downregulation of another transcription factor, the precise mechanism of *EWS/FLI1* regulation needs to be elucidated. Nonetheless, the reduction in EWS/FLI1 in addition to the decrease in FoxM1 suggests that thiostrepton does efficiently block both oncoproteins, thus providing an important added value to its efficacy for the treatment of EWS.

Using a nude mouse xenograft system, we show that thiostrepton efficiently decreases the tumorigenicity of EWS cells, evidenced by the significant delay in tumor growth as reflected by the much smaller (~5-fold) tumor volumes attained in thiostrepton-treated animals relative to vehicle-treated controls (Fig. 4). Our data also reveal that an important factor in the mechanism of action of thiostrepton on the proliferation EWS cells in culture and the decreased tumorigenicity *in vivo* is the activation of a caspase-dependent apoptotic response primarily involving caspase 7 as the main cell death effector (Fig. 5). In this regard, our results on the effect of thiostrepton on EWS cells seems to differ from data in previous reports indicating the predominant involvement of caspases 3, 8 or 9 in the apoptotic processes promoted by thiostrepton treatment in human cell lines derived from a variety of other tumor types (29,43–46). However, our results agree with previous findings indicating that caspase 7 activation mediated EWS cell death after exposure to other anticancer agents (31). Taking together our results from EWS cell cultures and from tumorigenicity assays, it appears that the action of thiostrepton on EWS involves an effect on the cell cycle, by arresting the cells at the G1 and G2/M phases of the cell cycle, and ultimately triggering cell death.

Because overexpression of FoxM1 has been demonstrated to lead to deregulated cell growth, and FoxM1 has been found being overexpressed in many cancers, it has been frequently argued that targeting FoxM1 may provide an opportunity towards curbing tumorigenesis. In fact, it was earlier proposed that FoxM1 may be the ‘Achilles’ heel’ of cancer (47). This notion is clearly supported by the wealth of information accumulated demonstrating that FoxM1 is involved essentially in most, if not all, processes associated with cancer progression (15,36). FoxM1 is a uniquely suitable target for therapeutic intervention in many tumor types. Our findings showing that thiostrepton is highly active *in vitro* and *in vivo* against EWS not only by inhibiting FoxM1, but also, and most importantly, by downregulating the expression of the EWS/FLI1 oncoprotein, identify thiostrepton as a novel therapeutic agent with dual inhibitory activity against EWS. Although the precise mechanism by which thiostrepton promotes EWS/FLI1 downregulation remains to be elucidated, results from the current study strongly suggest that thiostrepton alone may show greater efficacy in the treatment of EWS than against other tumor types, as it exerts its inhibitory action on EWS cells and tumors at concentrations lower than those reported to be required for effective antitumor activity on other cancer types (44,45,48,49). In addition, the use of thiostrepton in combination with other drugs also known to target EWS/FLI1 expression (50) may likely increase the therapeutic efficacy over currently available EWS treatments, thus improving the disease outcome for EWS patients.
